# Quantification of beam size impact on intensity-modulated proton therapy with robust optimization in head and neck cancer—comparison with intensity-modulated radiation therapy

**DOI:** 10.1093/jrr/rrae097

**Published:** 2024-12-27

**Authors:** Hiromi Baba, Kenji Hotta, Ryo Takahashi, Kana Motegi, Yuya Sugama, Takeji Sakae, Hidenobu Tachibana

**Affiliations:** Section of Radiation Safety and Quality Assurance, National Cancer Center Hospital East, 6-5-1 Kashiwanoha, Kashiwa, Chiba 277-8577, Japan; Doctoral Program in Biomedical Sciences, Graduate School of Comprehensive Human Sciences, University of Tsukuba, 1-1-1 Tennoudai, Tsukuba, Ibaraki 305-8577, Japan; Section of Radiation Safety and Quality Assurance, National Cancer Center Hospital East, 6-5-1 Kashiwanoha, Kashiwa, Chiba 277-8577, Japan; Section of Radiation Safety and Quality Assurance, National Cancer Center Hospital East, 6-5-1 Kashiwanoha, Kashiwa, Chiba 277-8577, Japan; Section of Radiation Safety and Quality Assurance, National Cancer Center Hospital East, 6-5-1 Kashiwanoha, Kashiwa, Chiba 277-8577, Japan; Proton Therapy Center, Aizawa Hospital, 2-5-1 Honjo, Matsumoto, Nagano 390-8510, Japan; Doctoral Program in Biomedical Sciences, Graduate School of Comprehensive Human Sciences, University of Tsukuba, 1-1-1 Tennoudai, Tsukuba, Ibaraki 305-8577, Japan; Section of Radiation Safety and Quality Assurance, National Cancer Center Hospital East, 6-5-1 Kashiwanoha, Kashiwa, Chiba 277-8577, Japan

**Keywords:** intensity-modulated proton therapy, head and neck cancer, beam size, robustness, robust optimization

## Abstract

We assessed the effect of beam size on plan robustness for intensity-modulated proton therapy (IMPT) of head and neck cancer (HNC) and compared the plan quality including robustness with that of intensity-modulated radiation therapy (IMRT). IMPT plans were generated for six HNC patients using six beam sizes (air-sigma 3–17 mm at isocenter for a 70–230 MeV) and two optimization methods for planning target volume-based non-robust optimization (NRO) and clinical target volume (CTV)-based robust optimization (RO). Worst-case dosimetric parameters and plan robustness for CTV and organs-at-risk (OARs) were assessed under different scenarios, assuming a ± 1–5 mm setup error and a ± 3% range error. Statistical comparisons of NRO-IMPT, RO-IMPT and IMRT plans were performed. In regard to CTV-D99%, RO-IMPT with smaller beam size was more robust than RO-IMPT with larger beam sizes, whereas NRO-IMPT showed the opposite (*P* < 0.05). There was no significant difference in the robustness of the CTV-D99% and CTV-D95% between RO-IMPT and IMRT. The worst-case CTV coverage of IMRT (±5 mm/3%) for all patients was 96.0% ± 1.4% (D99%) and 97.9% ± 0.3% (D95%). For four out of six patients, the worst-case CTV-D95% for RO-IMPT (±1–5 mm/3%) were higher than those for IMRT. Compared with IMRT, RO-IMPT with smaller beam sizes achieved lower worst-case doses to OARs. In HNC treatment, utilizing smaller beam sizes in RO-IMPT improves plan robustness compared to larger beam sizes, achieving comparable target robustness and lower worst-case OARs doses compared to IMRT.

## INTRODUCTION

Robust optimization (RO) methods for intensity-modulated proton therapy (IMPT) planning have become mainstream worldwide. Clinically available RO methods in commercial treatment planning systems (TPSs) have been investigated for their ability to optimize the robustness of plans against variations in patient setup and range [1–3]. Several publications have shown that IMPT treatment plans generated using RO are more robust than those generated by planning target volume (PTV)-based non-robust optimization (NRO) [4–9]. Compared with intensity-modulated radiation therapy (IMRT), RO-IMPT has the capability to reduce the dose to organs-at-risk (OARs) by suppressing high-dose regions around the target and overall low-dose areas [10, 11].

In IMPT, the proton beam size is an important factor affecting the optimization of the conformal dose distribution. IMPT is performed with different beam sizes at each facility because the beam size differs according to the specification of the proton beam delivery system. For example, the air-sigma of the beam spot at one operational proton therapy facility was 5–15 mm, whereas at another facility, it was 2–6 mm [12]. A smaller beam size can achieve a more conformal dose distribution, thereby resulting in a reduced dose to OARs in IMPT plans [13–15]. Several reports showed that IMPT plans created with a larger beam size using NRO were highly robust for lung cancer [16–18]. Liu *et al.* evaluated plan robustness using RO-IMPT in lung cancer [12] and found no dependency on beam size with respect to the target. Rana *et al.* reported that small-beam-size plans using single-field optimization were more robust against setup and range uncertainties than plans with larger beam sizes in cases of lung cancer [19]. It has also been reported that by using the repainting technique, the effects of interplay on plans with small and large beam sizes become comparable [12, 19]. However, to our knowledge, no study has addressed the impact of beam size, as a function of patient setup and proton range, on the treatment robustness of NRO-IMPT and RO-IMPT with multi-field optimization in head and neck cancer (HNC). Furthermore, there are few reports comparing the robustness of IMRT and RO-IMPT, yet RO-IMPT is considered capable of reducing OAR doses [9, 20].

It is unclear whether claims of greater robustness for RO-IMPT are justified. This means that the RO method may not be clinically verified because there is no threshold value to ensure a plan is clinically robust against variations in range and patient setup for proton beam therapy and in patient setup for X-ray radiotherapy. In contrast, there is a long history of IMRT in HNC, and we believe that 3–5 mm setup margin may be clinically feasible for the robustness from the clinical outcome [21–24]. Thus, RO-IMPT should be compared with IMRT to create clinically robust criteria. Additionally, the beam size affects the robustness of treatment and the quality of treatment plans. Because the beam sizes used for IMPT vary among proton therapy facilities, clinical outcomes may be facility-dependent. Therefore, quantification of the impact of beam size on IMPT concerning IMRT is necessary to conduct high-quality multicenter clinical trials and establish treatment planning goals for patients at each IMPT facility.

In this study, we analyzed the effect of beam size on the robustness of IMPT treatment plans for HNC, and evaluated plan quality, focusing particularly on treatment uncertainty between IMPT and IMRT.

## MATERIALS AND METHODS

### Patient data

Six patients with HNC and cervical lymph node metastasis who received IMRT at our institution in 2014–2016 were randomly selected for the analysis (Table 1). This study was approved by the institutional review board of the institution (approval number 2017–440). The treatment planning protocol is described in detail in Supplement 1. Both IMRT and IMPT plans were optimized to deliver the prescribed dose to the target volumes and satisfy the OAR dose constraints of our institution while minimizing doses to the OARs.

**Table 1:** Summary of the patients included in this study. CTV, clinical target volume

						CTV (cm^3^)		
Patient No	Gender	Age	Site	Clinical / pathological TNM stage	Prescription Dose (GyRBE) / fraction	High	Inter	Low
1	Male	61	oropharynx	pT4a N2c M0	70/ 60/ 54/ 33fx	640.3	45.2	47.2
2	Male	53	oropharynx	pT1 N2b M0	70/ 60/ 54/ 33fx	178.3	48.4	121.4
3	Male	62	oropharynx	pT4a N2b M0	70/ 60/ 54/ 33fx	289.0	105.5	41.0
4	Female	68	oropharynx	pT1 N2b M0	70/ 60/ 54/ 33fx	100.0	7.3	105.4
5	Male	61	hypopharynx	pT2 N2c M0	70/ 60/ 56/ 35fx	155.4	100.7	27.5
6	Male	62	hypopharynx	pT3 N2c M0	70/ 60/ 56/ 35fx	244.7	93.7	22.2

### IMRT treatment planning

**Fig. 1:**
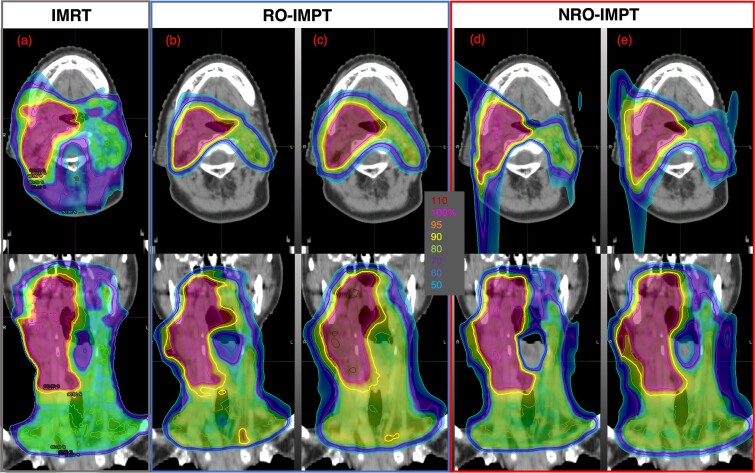
Dose distributions for Patient 4 in a nominal case (0 mm/0% errors). (a) IMRT plan. (b) RO-IMPT plan using a beam spot size of S−. (c) RO-IMPT plan using a beam spot size of L+. (d) NRO-IMPT plan using a beam spot size of S−. (e) NRO-IMPT plan using a beam spot size of L+. The upper and lower rows show transverse and coronal views, respectively. IMRT, intensity-modulated radiation therapy; IMPT, intensity-modulated proton therapy; RO, robust optimization; NRO, non-robust optimization; S−/L+, air-sigma of 3–8 mm / 6–17 mm at isocenter.

IMRT plans were generated using the step-and-shoot technique with a Pinnacle^3^ TPS (Philips Medical Systems, Milpitas, CA, USA). Each PTV was generated via a 5 mm isotropic expansion of the corresponding CTV (Fig. 1a).

### IMPT treatment planning

**Fig. 2:**
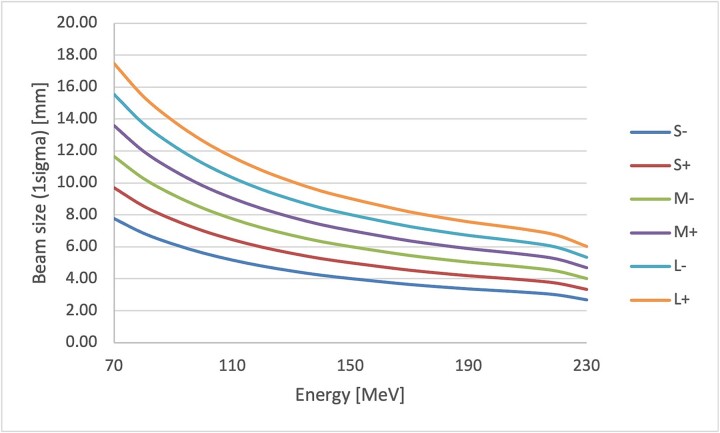
Beam size (in-air sigma at the isocenter) as a functional of energy for a line-scanning beam with six evenly spaced beam sizes. S−/S+/M−/M+/L−/L+, air-sigma of 3–8 mm / 3–10 mm / 4–12 mm / 5–14 mm / 5–16 mm / 6–17 mm at isocenter.

IMPT plans were generated using an Eclipse TPS (Varian Medical Systems, Palo Alto, CA, USA) with a relative biological effectiveness (RBE) of 1.1. Two optimization approaches were used: PTV-based NRO methods and CTV-based RO methods. The RO calculation method used Worst-case scenario optimization and Selective RO [2, 3]. Three fields (60°, 300° and 180° ±30°) were used, with a range shifter applied in two of them. The same gantry angle was used for NRO and RO-IMPT. Beam modeling was performed for the universal nozzle beam (Sumitomo Heavy Industries, Niihama, Ehime, Japan). Based on the commissioning data, a line-scanning beam with six evenly spaced beam sizes was used with the smallest beam size set by referencing the lower limit established for the modeling nozzle (Fig. 2). The air-sigma of the 70–230 MeV beam spot was either 3–8 mm (S−), 3–10 mm (S+), 4–12 mm (M−), 5–14 mm (M+), 5–16 mm (L−) or 6–17 mm (L+) at isocenter. The dose calculation algorithm used Proton Convolution Superposition. The calculation grid size was set to 2.5 × 2.5 × 3.0 mm, which is used in our facility's clinical practice. Each PTV for the NRO-IMPT plans was generated via a 5 mm isotropic expansion of the corresponding CTV (Fig. 1d,e). PTVs were not used for the RO-IMPT plans, but 12 scenarios were considered for the CTVs during the optimization process, incorporating the combination of a ± 5 mm setup uncertainty and ± 3% range uncertainty in the right–left, anterior–posterior and superior–inferior directions (Fig. 1b,c).

### Multi-case scenarios

To assess the effect of beam size on IMPT plan quality, a multi-case dose–volume histogram (DVH) was calculated assuming treatment uncertainty involving combinations of ±1, ±2, ±3, ±4 or ± 5 mm setup errors with ±3% range errors. In this calculation, the isocenter was shifted along the right–left, anterior–posterior and superior–inferior directions. Sixty-one dose distributions corresponding to the multi-case scenarios were created, including a nominal case (0 mm/0% errors).

### Evaluation

Doses delivered to 2%, 95% and 99% (D2%, D95% and D99%, respectively) of the high-risk CTV, D2% of spinal cord, D2% of brainstem and the mean dose (Dmean) delivered to each of the ipsilateral-parotid gland, contralateral-parotid gland, oral cavity and larynx were evaluated from the multi-case DVHs. The dosimetric parameters, including dose coverage of the CTVs and dose sparing of OARs in RO-IMPT plans, were compared with those in NRO-IMPT and IMRT plans.

To reveal plan robustness, the bandwidth was estimated from the difference between the maximum and minimum value of the dosimetric parameters of the multi-case DVHs (±5 mm / ±3%). Box-and-whisker plots were used to show the bandwidth for the six patients. When the median values of the bandwidth were smaller, the plan approach was more robust [5]. A smaller interquartile range indicates a plan that is more robust with respect to inter-patient variability. To clearly demonstrate the effect of beam size on plan robustness for each plan approach, representative values of the minimum beam size (S−) and maximum beam size (L+) were compared with the bandwidth.

To demonstrate plan quality, including robustness, the doses to the target and OARs in the worst-case scenario, defined as that with the lowest dose to the target coverage and the highest dose to the OARs, were compared [25].

### Statistical analyses

Statistical analyses were conducted with the guidance and consultation of a professional statistician to ensure the robustness and accuracy of the results. Pair-wise comparisons of IMRT and IMPT for CTVs and OARs to examine the effects of beam size differences were performed using the Wilcoxon signed-rank test, and the Friedman test was used to compare more than two groups. A *P* value <0.05 was considered statistically significant.

## RESULTS

### Effect of beam size on plan robustness

**Fig. 3:**
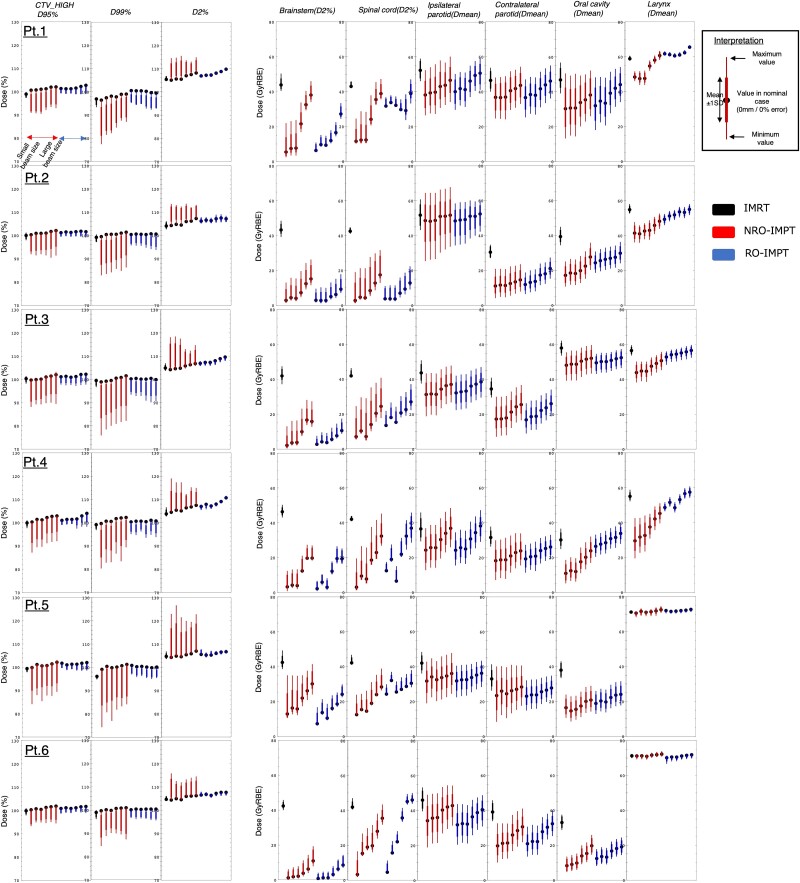
Comparison of dosimetric parameters, including clinical target volume (CTV) coverage and (organ at risk) OAR doses, for NRO-IMPT, RO-IMPT and IMRT plans for all patients. Nominal plan quality (circles) and its fluctuation (lines) in multi-case scenarios are shown. For each parameter, the data are displayed from left to right in order of ascending beam size. D2%, D95% and D99% denote the doses delivered to 2%, 95% and 99% of the target volume, respectively, and Dmean represents the mean dose delivered to the target volume.

**Fig. 4:**
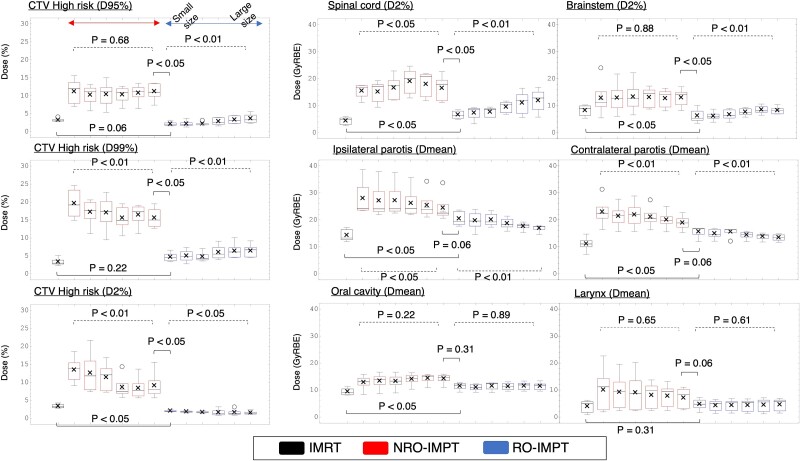
Box-and-whisker plots of the bandwidth for each patient, calculated from the difference between the maximum and minimum dose delivered to the CTV and OARs in multi-case scenarios (5 mm setup error/3% range error). The center line and the cross in each box represent the median and mean values, respectively. The upper and lower edges of each box represent the first and third quartiles, respectively, and values outside 1.5× the interquartile range are marked as outliers (circles). Smaller median values of the bandwidth indicate a more robust plan. A smaller interquartile range indicates a plan that is more robust with respect to inter-patient variability. The *P* values of the Friedman test (dotted line) and the Wilcoxon signed-rank test (solid line) are shown. Full statistical results of the Wilcoxon signed-rank test are presented in Supplement 2.

For each patient, multi-case DVH index for patient-specific CTVs and OARs, assuming a ± 5 mm setup error and ± 3% range error, are illustrated in Fig. 3. It displays the nominal-case doses for IMRT, NRO-IMPT and RO-IMPT plans, including the dose variations (mean ± 1 standard deviation) and the worst-case doses (maximum and minimum values) for each scenario. Using these data, we compared plan robustness according to the bandwidth method, using the multi-case DVHs of the CTVs and OARs (Fig. 4). All *P* values are shown in Supplement 2. The median, mean and interquartile range of the CTVs for RO-IMPT were smaller than those for NRO-IMPT (*P* < 0.05).

We compared RO-IMPT with S− to NRO-IMPT with L+. The DVH bandwidths of all OARs except both parotid glands, the oral cavity and the larynx were smaller for RO-IMPT with S− than those for NRO-IMPT with L+ and assumed to be the most robust (median differences: CTV [D95%], 9.00%; CTV [D99%], 9.40%; CTV [D2%], 5.75%, brainstem [D2%], 7.70 GyRBE; spinal cord [D2%], 9.11 GyRBE; *P* < 0.05). Thus, RO-IMPT was more robust than NRO-IMPT with regard to CTV coverage, regardless of beam size.

We compared RO-IMPT with S− to NRO-IMPT with S−. The DVH bandwidths all OARs except the oral cavity and larynx were smaller for RO-IMPT with S− than those for NRO-IMPT with S− (median differences: brainstem [D2%], 6.44 GyRBE; spinal cord [D2%], 9.67 GyRBE; ipsilateral parotid [Dmean], 5.10 GyRBE; contralateral parotid [Dmean], 5.66 GyRBE; *P* < 0.05).

We compared RO-IMPT with L+ to NRO-IMPT with L+. The DVH bandwidths of all OARs except the brainstem and spinal cord were smaller for RO-IMPT with L+ than those for NRO-IMPT with L+ (median differences: ipsilateral parotid [Dmean], 5.17 GyRBE; contralateral parotid [Dmean], 5.44 GyRBE; oral cavity [Dmean], 3.13 GyRBE; larynx [Dmean], 2.36 GyRBE; *P* < 0.05).

We compared RO-IMPT with S− to RO-IMPT with L+. For RO-IMPT with S−, the DVH bandwidths of the CTV (D99% and D95%) were smaller than those for RO-IMPT with L+ (median differences: CTV [D95%], 1.60%; CTV [D99%], 1.80%; *P* < 0.05). However, the DVH bandwidths of CTV (D2%) and both parotid glands for RO-IMPT with L+ were smaller than those for RO-IMPT with S− (median differences: CTV [D2%], 0.70%; ipsilateral parotid [Dmean], 2.18 GyRBE; contralateral parotid [Dmean], 2.94 GyRBE; *P* < 0.05).

We compared NRO-IMPT with S− to NRO-IMPT with L+. For NRO-IMPT with L+, the DVH bandwidths of the CTV (D99% and D2%) and contralateral parotid gland were smaller than those for NRO-IMPT with S− (median differences: CTV [D99%], 4.80%; CTV [D2%], 4.90%; contralateral parotid [Dmean], 3.16 GyRBE; *P* < 0.05).

We compared RO-IMPT to IMRT. The DVH bandwidths of the spinal cord, both parotid glands, and the oral cavity for RO-IMPT with S− were larger than those for IMRT (median differences: spinal cord [Dmean], 1.72 GyRBE; ipsilateral parotid [Dmean], 6.67 GyRBE; contralateral parotid [Dmean], 5.49 GyRBE, oral cavity [Dmean], 2.40 GyRBE; *P* < 0.05). In contrast, the DVH bandwidths of the CTV (D2%) and brainstem were smaller for RO-IMPT with S− than those for IMRT (median differences: CTV [D2%], 1.15%; brainstem [D2%], 4.00 GyRBE; *P* < 0.05). The DVH bandwidths of the ipsilateral parotid gland and oral cavity for RO-IMPT with L+ were larger than those for IMRT (median differences: ipsilateral parotid [Dmean], 4.49 GyRBE; oral cavity [Dmean], 2.17 GyRBE; *P* < 0.05). The target robustness of the CTV (D95%) and CTV (D99%) for RO-IMPT were not significantly different from those for IMRT.

### Comparison of plan quality in the worst-case scenario

The median values of the worst-case CTV coverage of IMRT with a 5 mm PTV margin, assuming a ± 5 mm/3% error for all patients, were 96.6% (D99%) and 98.0% (D95%). Table 2 shows the number of NRO- and RO-IMPT plans (±1–5 mm/±3%) that resulted in a worst-case dose higher than that of the corresponding IMRT plan assuming ±5 mm/3% error. The worst-case doses for each plan approaches are shown in Supplement 3. The CTV (D99%) values for all RO-IMPT plans with S− were higher than those for the corresponding IMRT plans when the setup errors were ± 1–3 mm. There was at least one RO-IMPT plan that had a CTV (D99%) value higher than that of the corresponding IMRT plan when the setup errors were ± 4–5 mm. Additionally, for four out of six patients, the CTV (D95%) values for RO-IMPT were higher than those for IMRT when the isocenter shifts were ± 1–5 mm. The worst-case CTV (D99%) increased as the beam size was reduced for RO-IMPT and conversely decreased for NRO-IMPT (median: RO (S−), 95.6%; RO (L+), 93.9%; NRO (S−), 79.0%; NRO (L+), 86.8%; *P* < 0.05). The worst-case target coverages for the NRO-IMPT plans with beam sizes of L− to L+ (D99%) and M+ to L+ (D95%) were higher than those for the corresponding IMRT plans when the isocenter shifts were ± 1 mm.

**Table 2:** Number of NRO- and RO-IMPT plans (±1–5 mm/3% error) in which worst-case scenario target coverage was higher than that of the corresponding IMRT plan (±5 mm/3% error). The numbers presented range from a minimum of 0 to a maximum of 6, with a value of 6 indicating that target coverage of IMPT plans for all patients was higher than that of IMRT plan assuming ±5 mm/3% error. NRO, non-robust optimization; RO, robust optimization; IMPT, intensity-modulated proton therapy; IMRT, intensity-modulated radiation therapy

		NRO-IMPT						RO-IMPT					
	Setup error / Range error	S−	S+	M−	M+	L−	L+	S−	S+	M−	M+	L−	L+
CTV_HIGH D99%	±1 mm / ±3%	0	0	0	0	1	1	6	6	6	6	5	5
	±2 mm / ±3%	0	0	0	0	0	0	6	6	6	5	5	4
	±3 mm / ±3%	0	0	0	0	0	0	6	5	5	4	4	4
	±4 mm / ±3%	0	0	0	0	0	0	4	4	4	3	3	3
	±5 mm / ±3%	0	0	0	0	0	0	3	3	3	2	1	1
CTV_HIGH D95%	±1 mm / ±3%	0	0	0	2	3	2	6	6	6	6	6	6
	±2 mm / ±3%	0	0	0	0	0	0	6	6	6	6	6	6
	±3 mm / ±3%	0	0	0	0	0	0	6	6	6	6	6	6
	±4 mm / ±3%	0	0	0	0	0	0	6	6	6	6	6	6
	±5 mm / ±3%	0	0	0	0	0	0	5	5	5	4	5	5

**Fig. 5:**
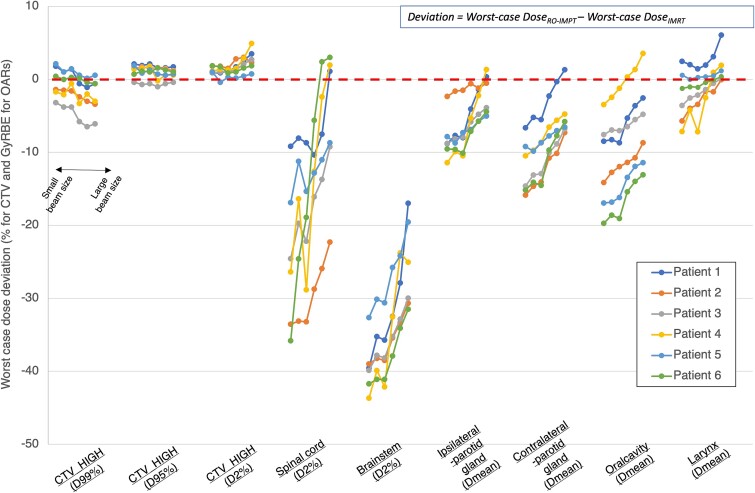
Dose deviation between RO-IMPT and IMRT plans for each worst-case scenario (±5 mm/3% error). A dose deviation below zero (dotted line) indicates that RO-IMPT resulted in a lower worst-case dose to OAR than IMRT. For D99% (CTV) and D95% (CTV), a dose deviation greater than or equal to zero indicates that RO-IMPT had a superior worst-case target coverage than IMRT. For each parameter, the data are displayed from left to right in order of ascending beam size.

Figure 5 compares dose deviations of RO-IMPT against IMRT for the worst-case scenario (±5 mm/3%) of each patient. For the CTV, the median dose deviations of RO-IMPT with S− relative to those of IMRT were as follows: CTV (D99%), −0.49%; CTV (D95%), +1.45%; CTV (D2%), +1.06%. For CTV (D95%) and CTV (D2%), RO-IMPT resulted in a higher worst-case dose than IMRT for most patients. A dose deviation below zero indicates that the worst-case OAR dose was smaller for RO-IMPT than for IMRT. For all OARs, the dose deviations increased as the beam size was reduced. The maximum worst-case dose deviations between RO-IMPT plans with beam sizes of S− and IMRT in all patients were as follows: brainstem [D2%], 43.7 GyRBE; spinal cord [D2%], 35.8 GyRBE; ipsilateral parotid [Dmean], 11.4 GyRBE; contralateral parotid [Dmean], 15.9 GyRBE; oral cavity [Dmean], 19.7 GyRBE; larynx [D2%], 7.1 GyRBE. With a beam size of M− or less, the worst-case dose to all OARs (except the larynx) was smaller for RO-IMPT than that for IMRT for all patients.

## DISCUSSION

For six patients with HNC, we evaluated the robustness of IMPT plans with and without RO approaches using six different beam sizes. We performed the RO-IMPT optimization for target coverage and OAR dose reduction in multiple scenarios combining setup and range uncertainties. In contrast, the optimization of NRO-IMPT plans assumed a single scenario (i.e. a nominal case with 0 mm/0% errors) that did not include robustness across multiple scenarios. RO-IMPT showed higher robustness in regard to target coverage when the beam size became smaller, whereas NRO-IMPT showed the opposite. The use of a small beam size in RO-IMPT results in a high degree of flexibility for creating a satisfactory plan, the potential to create a more complicated dose distribution with better OAR sparing, and plan robustness.

As shown in Fig. 4, plans with smaller beam sizes exhibited greater robustness in target coverage, spinal cord and brainstem. Liu *et al.* examined plan robustness using RO-IMPT with small and large beam sizes in lung cancer [12] and found no statistically significant difference in robustness of target coverage between small and large beam sizes. However, they evaluated the setup and range uncertainties separately. In contrast, Rana *et al.* also reported plan robustness with a combination of setup and range uncertainties in lung cancer [19]. Consistent with our findings, their results showed that small beam sizes were more robust against setup and range uncertainties than large beam sizes. Additionally, Kraan *et al.* reported that plans created using small beam sizes exhibit high robustness against beam size variations since the commissioning of a proton delivery system [26]. Smaller beam sizes are able to create more robust target-coverage plans not only against patient-derived uncertainties, such as setup and range errors, but also against machine-derived uncertainties. On the other hand, smaller beam sizes may result in longer irradiation times. This is because smaller beam sizes generally involve more spots and layers than larger ones. In lung cancer cases, Liu *et al.* reported that irradiation times for smaller beam sizes were, on average, 28% longer than those for larger beam sizes [12]. It is important to note that longer field irradiation times may increase intra-fractional randomization, potentially leading to increased setup errors, which requires careful consideration.

It is evident that setup uncertainty is closely associated with clinical outcomes in radiation therapy. In PTV-based IMRT planning, which is the standard treatment for HNC, the margin covers assumed setup fluctuation from the nominal plan to obtain robustness in the target coverage [27, 28]. For IMRT, the nominal plan is designed to meet the DVH criteria, but plan robustness is generally not evaluated. For RO-IMPT, the plan robustness is commonly evaluated and DVH criteria for the CTV are established not only for the nominal case but also the worst case. However, these criteria are not standardized and may vary among facilities. In our study, we created plans for each beam size that met at least the DVH criteria for nominal-case CTV coverage (D99% > 98% and D2% < 110%). For each beam size and setup error, we compared RO-IMPT plans with IMRT plans in terms of worst-case doses to the CTV and OARs. Figure 4 shows that the target robustness for CTV-based RO-IMPT and PTV-based IMRT were not significantly different. Similarly, in cases of skull-based chordomas, Noufal *et al.* reported that the robustness of CTV (D95%) in RO-IMPT was equivalent to that in standard IMRT [20]. On the other hands, the worst-case CTV coverage of RO-IMPT in HNC cases in this study depended on the beam size and setup error, even with the DVH criteria for nominal-case CTV coverage (Table 2, Supplement 3). Of course, the errors in setup and beam range vary across different facilities because of different patient immobilization and image-guidance systems and also different beam sizes from different proton delivery systems. Because the plan robustness of RO-IMPT varies depending on proton delivery system and patient immobilization system, medical physicists should understand the plan quality and robustness specific to their facility and establish the robustness criteria before starting HNC treatment. Additionally, Fig. 5 indicates that for patient referral to IMPT, robust evaluation should be taken into account when comparing IMPT with IMRT rather than relying only on nominal plans. Furthermore, we assert that inter-institutional differences in the plan quality and robustness need to be understood during the credentialing phase of multi-institutional clinical trials of IMPT for HNC. This is because differences in the worst-case OAR doses among facilities may occur even if the planned CTVs meet the DVH criteria in the worst-case scenario.

Our results indicate that RO-IMPT for HNC using a 70–230 MeV beam with an air-sigma of 4–12 mm at isocenter results in comparable target robustness and a lower worst-case OAR dose than IMRT (Fig. 5). The dose distribution of RO-IMPT can be influenced not only by the beam size but also by other factors, such as beam angles and patient characteristics. The inclination of the beam size from low to high energy varies depending on the nozzle structure and transport structure. Additionally, low- and medium-energy beams should often be chosen when treating HNC because the target is located at a shallow tissue depth. Thus, on the basis of our findings, it is advisable to use a target beam size with an air-sigma of 6 mm or less at isocenter for a 150 MeV beam when performing head-and-neck RO-IMPT, assuming the same beam characteristics (e.g. range shifter) and patient characteristics.

## PRESENTATION AT A CONFERENCE

None declared.

## Supplementary Material

Supplement_1_Clean_rrae097

Supplement_2_rrae097

Supplement_3_rrae097

## References

[1] Fredriksson A, Forsgren A, Hårdemark B. Minimax optimization for handling range and setup uncertainties in proton therapy. Med Phys 2011;38:1672–84. 10.1118/1.3556559. 21520880

[2] Liu W, Zhang X, Li Y, Mohan R. Robust optimization of intensity modulated proton therapy. Med Phys 2012;39:1079–91. 10.1118/1.3679340. 22320818 PMC3281975

[3] Li Y, Niemela P, Liao L et al. Selective robust optimization: a new intensity-modulated proton therapy optimization strategy. Med Phys 2015;42:4840–7. 10.1118/1.4923171. 26233211

[4] Liu W, Frank SJ, Li X et al. Effectiveness of robust optimization in intensity-modulated proton therapy planning for head and neck cancers. Med Phys 2013;40:051711. 10.1118/1.4801899. 23635259 PMC3651255

[5] Liu W, Frank SJ, Li X et al. PTV-based IMPT optimization incorporating planning risk volumes vs robust optimization. Med Phys 2013;40:021709. 10.1118/1.4774363. 23387732 PMC3562272

[6] van Dijk LV, Steenbakkers RJ, ten Haken B et al. Robust intensity modulated proton therapy (IMPT) increases estimated clinical benefit in head and neck cancer patients. PLoS One 2016;11:e0152477. 10.1371/journal.pone.0152477. 27030987 PMC4816406

[7] Cubillos-Mesías M, Baumann M, Troost EGC et al. Impact of robust treatment planning on single- and multi-field optimized plans for proton beam therapy of unilateral head and neck target volumes. Radiat Oncol 2017;12:190. 10.1186/s13014-017-0931-8. 29183377 PMC5706329

[8] Unkelbach J, Bortfeld T, Martin BC et al. Reducing the sensitivity of IMPT treatment plans to setup errors and range uncertainties via probabilistic treatment planning. Med Phys 2009;36:149–63. 10.1118/1.3021139. 19235384 PMC2673668

[9] Stuschke M, Kaiser A, Pöttgen C et al. Potentials of robust intensity modulated scanning proton plans for locally advanced lung cancer in comparison to intensity modulated photon plans. Radiother Oncol 2012;104:45–51. 10.1016/j.radonc.2012.03.017. 22560714

[10] Stuschke M, Kaiser A, Abu-Jawad J et al. Re-irradiation of recurrent head and neck carcinomas: comparison of robust intensity modulated proton therapy treatment plans with helical tomotherapy. Radiat Oncol 2013;8:93. 10.1186/1748-717X-8-93. 23601204 PMC3648492

[11] Stuschke M, Kaiser A, Abu Jawad J et al. Multi-scenario based robust intensity-modulated proton therapy (IMPT) plans can account for set-up errors more effectively in terms of normal tissue sparing than planning target volume (PTV) based intensity-modulated photon plans in the head and neck region. Radiat Oncol 2013;8:145. 10.1186/1748-717X-8-145. 23773560 PMC3695849

[12] Liu C, Schild SE, Chang JY et al. Impact of spot size and spacing on the quality of robustly optimized intensity modulated proton therapy plans for lung cancer. Int J Radiat Oncol Biol Phys 2018;101:479–89. 10.1016/j.ijrobp.2018.02.009. 29550033 PMC5935576

[13] Steneker M, Lomax A, Schneider U. Intensity modulated photon and proton therapy for the treatment of head and neck tumors. Radiother Oncol 2006;80:263–7. 10.1016/j.radonc.2006.07.025. 16916557

[14] van de Water TA, Lomax AJ, Bijl HP et al. Using a reduced spot size for intensity-modulated proton therapy potentially improves salivary gland-sparing in oropharyngeal cancer. Int J Radiat Oncol Biol Phys 2012;82:e313–9. 10.1016/j.ijrobp.2011.05.005. 21708427

[15] Moteabbed M, Yock TI, Depauw N et al. Impact of spot size and beam-shaping devices on the treatment plan quality for pencil beam scanning proton therapy. Int J Radiat Oncol Biol Phys 2016;95:190–8. 10.1016/j.ijrobp.2015.12.368. 27084640 PMC4834139

[16] Sio TT, Merrell KW, Beltran CJ et al. Spot-scanned pancreatic stereotactic body proton therapy: a dosimetric feasibility and robustness study. Phys Med 2016;32:331–42. 10.1016/j.ejmp.2015.12.009. 26883369

[17] Dowdell S, Grassberger C, Sharp GC et al. Interplay effects in proton scanning for lung: a 4D Monte Carlo study assessing the impact of tumor and beam delivery. Phys Med Biol 2013;58:4137–56. 10.1088/0031-9155/58/12/4137. 23689035 PMC3752993

[18] Grassberger C, Dowdell S, Lomax A et al. Motion interplay as a function of patient parameters and spot size in spot scanning proton therapy for lung cancer. I nt J Radiat Oncol Biol Phys 2013;86:380–6. 10.1016/j.ijrobp.2013.01.024. PMC3646997 23462423

[19] Rana S, Rosenfeld AB. Small spot size versus large spot size: effect on plan quality for lung cancer in pencil beam scanning proton therapy. J Appl Clin Med Phys 2022;23:e13512. 10.1002/acm2.13512. 34989458 PMC8833272

[20] Noufal MP, Widesott L, Sharma SD et al. The role of plan robustness evaluation in comparing protons and photons plans—an application on IMPT and IMRT plans in skull base chordomas. J Med Phys 2020;45:206–14. 10.4103/jmp.JMP_45_20. 33953495 PMC8074721

[21] Lambrecht M, Nevens D, Nuyts S. Intensity-modulated radiotherapy vs. parotid-sparing 3D conformal radiotherapy. Effect on outcome and toxicity in locally advanced head and neck cancer. Strahlenther Onkol 2013;189:223–9. 10.1007/s00066-012-0289-7. 23319256

[22] Gupta T, Agarwal J, Jain S et al. Three-dimensional conformal radiotherapy (3D-CRT) versus intensity modulated radiation therapy (IMRT) in squamous cell carcinoma of the head and neck: a randomized controlled trial. Radiother Oncol 2012;104:343–8. 10.1016/j.radonc.2012.07.001. 22853852

[23] Toledano I, Graff P, Serre A et al. Intensity-modulated radiotherapy in head and neck cancer: results of the prospective study GORTEC 2004-03. Radiother Oncol 2012;103:57–62. 10.1016/j.radonc.2011.12.010. 22296746

[24] Eisbruch A, Harris J, Garden AS et al. Multi-institutional trial of accelerated hypofractionated intensity-modulated radiation therapy for early-stage oropharyngeal cancer (RTOG 00-22). Int J Radiat Oncol Biol Phys 2010;76:1333–8. 10.1016/j.ijrobp.2009.04.011. 19540060 PMC2846217

[25] Lomax AJ, Boehringer T, Coray A et al. Intensity modulated proton therapy: a clinical example. Med Phys 2001;28:317–24. 10.1118/1.1350587. 11318312

[26] Kraan AC, Depauw N, Clasie B et al. Impact of spot size variations on dose in scanned proton beam therapy. Phys Med 2019;57:58–64. 10.1016/j.ejmp.2018.12.011. 30738532

[27] International Commission on Radiation Units and Measurements . Prescribing, Recording, and Reporting Photon Beam Therapy (Supplement to ICRU Report 50). ICRU Report 62. Bethesda. USA, 1999.

[28] Stroom JC, Heijmen BJ. Geometrical uncertainties, radiotherapy planning margins, and the ICRU-62 report. Radiother Oncol 2002;64:75–83. 10.1016/S0167-8140(02)00140-8. 12208578

