# Supplementary material for: Quantification of beam size impact on intensity-modulated proton therapy with robust optimization in head and neck cancer—comparison with intensity-modulated radiation therapy
Source: J Radiat Res. 2024 Dec 27;66(1):65–73. doi: 10.1093/jrr/rrae097 (PMC11753836; doi:10.1093/jrr/rrae097)
Supplement: Supplement_1_Clean_rrae097 [file supplement_1_clean_rrae097.pdf]

Supplement 1: Treatment planning

Planning CT

Planning CT scans were performed with 3-mm slice thickness in the supine position using Aquilion-ONE scanners (Canon Medical Systems, Otawara, Tochigi, Japan).

Contouring

The high-risk clinical target volume (CTV), which included the primary tumor and clinically metastatic or potentially metastatic lymph nodes, was expanded to the elective lymph nodes to generate intermediate and low-risk CTVs. The gross tumor volume (GTV), CTV, PTV, and OARs, including spinal cord, brainstem, parotid gland, oral cavity, and larynx, were delineated by a radiation oncologist. Patient 1 had a bilateral high-risk CTV in the cervical lymph node area, whereas the other patients had unilateral high-risk CTVs and inter- or low-risk CTVs in the contralateral neck. The simultaneous integrated boost (SIB) method was used, and the prescribed dose was 70 GyRBE to the high-risk CTV, 60 GyRBE to the intermediate-risk CTV, and 54 or 56 GyRBE to the low-risk CTV in 33 or 35 fractions.

IMRT planning

IMRT plans were used for clinical treatments. Our dosimetrists and oncologists generated the plans using the step-and-shoot technique with a Pinnacle^3 TPS (Philips Medical Systems, Milpitas, CA, USA). Nine 6-MV X-ray fields were used in the PTV-based IMRT plans. The calculation grid size was set to 2.0 × 2.0 × 3.0 mm, which is used in our facility's clinical practice. Each PTV was generated via a 5-mm isotropic expansion of the corresponding CTV.

IMPT planning

IMPT plans were generated using an Eclipse TPS (Varian Medical Systems, Palo Alto, CA, USA) with a relative biological effectiveness (RBE) of 1.1. In creating the IMPT plans, a different planner from the one who developed the IMRT plans was responsible for the task, and the planner was blinded with no dose information about the IMRT plans. Two optimization approaches were used: PTV-based NRO methods and CTV-based RO methods. The RO calculation method used Worst-case scenario optimization and Selective robust optimization. Three fields (60°, 300°, and 180° ±30°) were used, and entering through OARs and regions of inhomogeneity was avoided. The same gantry angle was used for NRO and RO-IMPT in each patient. Beam modeling was performed for the universal nozzle beam (Sumitomo Heavy Industries, Niihama, Ehime, Japan). Based on

the commissioning data, a line-scanning beam with six evenly spaced beam sizes was used with the smallest beam size set by referencing the lower limit established for the modeling nozzle. The beam size-dependent in-air energies were sigma 3–8 mm (S−), 3–10 mm (S+), 4–12 mm (M−), 5–14 mm (M+), 5–16 mm (L−) and 6–17 mm (L+) at isocenter for a 70–230 MeV range. The dose calculation algorithm used Proton Convolution Superposition. The calculation grid size was set to 2.5 × 2.5 × 3.0 mm, which is used in our facility's clinical practice. The spot spacings for each beam size were calculated automatically by the TPS. The range shifter had a 4.11-cm water-equivalent thickness in total. Each PTV for the NRO-IMPT plans was generated via a 5-mm isotropic expansion of the corresponding CTV. PTVs were not used for the RO-IMPT plans, but 5-mm setup uncertainty and 3% range uncertainty were used for the CTVs during the optimization process. Both IMRT and IMPT plans were optimized to deliver the prescribed dose to the target volumes (RO-IMPT: $D99\%_{CTVs} \geq 98\%$ and $D2\%_{CTVs} \leq 110\%$ of the prescribed dose, NRO-IMPT and IMRT: $D95\%_{PTVs} \geq 95\%$ and $D2\%_{PTVs} \leq 110\%$ of the prescribed dose) and achieve the OAR dose constraints of our institution while sparing the OARs as much as possible.
