# Supplementary material for: Quantification of beam size impact on intensity-modulated proton therapy with robust optimization in head and neck cancer—comparison with intensity-modulated radiation therapy
Source: J Radiat Res. 2024 Dec 27;66(1):65–73. doi: 10.1093/jrr/rrae097 (PMC11753836; doi:10.1093/jrr/rrae097)
Supplement: Supplement_2_rrae097 [file supplement_2_rrae097.pdf]

Supplement 2

P values of Wilcoxon signed-rank tests of the DVH band width in comparisons of IMRT, NRO-IMPT, and RO-IMPT plans. Statistically significant results (P < .05) are indicated in gray.

				NRO-IMPT						RO-IMPT					
			IMRT	S-	S+	M-	M+	L-	L+	S-	S+	M-	M+	L-	L+
CTV _HIGH (D95%)		IMRT		0.03	0.03	0.03	0.03	0.03	0.03	0.06	0.09	0.06	0.83	0.69	0.56
	NRO-IMPT	S-			0.22	0.34	0.22	0.44	1.00	0.03	0.03	0.03	0.03	0.03	0.03
		S+				0.92	0.67	0.84	0.31	0.03	0.03	0.03	0.03	0.03	0.03
		M-					0.84	1.00	0.44	0.03	0.03	0.03	0.03	0.03	0.03
		M+						0.67	0.11	0.03	0.03	0.03	0.03	0.03	0.03
		L-							0.40	0.03	0.03	0.03	0.03	0.03	0.03
		L+								0.03	0.03	0.03	0.03	0.03	0.03
	RO-IMPT	S-									0.84	1.00	0.09	0.04	0.03
		S+										0.56	0.16	0.03	0.03
		M-											0.09	0.03	0.03
		M+												0.04	0.04
		L-													0.09
		L+													
CTV _HIGH (D99%)		IMRT		0.03	0.03	0.03	0.03	0.04	0.03	0.22	0.09	0.09	0.06	0.06	0.06
	NRO-IMPT	S-			0.06	0.03	0.03	0.03	0.04	0.03	0.03	0.03	0.03	0.03	0.03
		S+				0.59	0.03	0.18	0.16	0.03	0.03	0.03	0.03	0.03	0.03
		M-					0.16	0.56	0.22	0.03	0.03	0.03	0.03	0.03	0.03
		M+						0.84	0.79	0.03	0.03	0.03	0.03	0.03	0.03
		L-							1.00	0.03	0.03	0.03	0.03	0.03	0.03
		L+								0.03	0.03	0.03	0.03	0.03	0.03
	RO-IMPT	S-									0.40	0.40	0.03	0.04	0.03
		S+										0.42	0.06	0.04	0.03
		M-											0.03	0.03	0.03
		M+												0.31	0.58
		L-													0.92
		L+													

				NRO-IMPT						RO-IMPT					
			IMRT	S-	S+	M-	M+	L-	L+	S-	S+	M-	M+	L-	L+
CTV _HIGH (D2%)		IMRT		0.03	0.03	0.03	0.03	0.03	0.03	0.03	0.03	0.03	0.06	0.06	0.03
	NRO-IMPT	S-			0.44	0.06	0.03	0.03	0.03	0.03	0.03	0.03	0.03	0.03	0.03
		S+				0.44	0.03	0.06	0.06	0.03	0.03	0.03	0.03	0.03	0.03
		M-					0.06	0.06	0.06	0.03	0.03	0.03	0.03	0.03	0.03
		M+						0.56	0.44	0.03	0.03	0.03	0.04	0.03	0.03
		L-							0.18	0.03	0.03	0.03	0.03	0.03	0.03
		L+								0.03	0.03	0.03	0.03	0.03	0.03
	RO-IMPT	S-									0.06	0.10	0.18	0.25	0.03
		S+										0.34	0.44	0.34	0.14
		M-											0.59	0.44	0.10
		M+												0.59	0.89
		L-													0.83
		L+													

				NRO-IMPT						RO-IMPT					
			IMRT	S-	S+	M-	M+	L-	L+	S-	S+	M-	M+	L-	L+
Spinal Cord (D2%)		IMRT		0.03	0.03	0.03	0.03	0.03	0.03	0.03	0.16	0.06	0.03	0.06	0.06
	NRO-IMPT	S-			1.00	0.22	0.03	0.16	0.84	0.03	0.03	0.03	0.04	0.09	0.11
		S+				0.22	0.06	0.44	0.84	0.03	0.03	0.03	0.03	0.03	0.06
		M-					0.06	0.83	0.31	0.03	0.03	0.03	0.03	0.03	0.03
		M+						0.09	0.03	0.03	0.03	0.03	0.03	0.03	0.03
		L-							0.16	0.03	0.03	0.03	0.03	0.06	0.06
		L+								0.03	0.03	0.03	0.03	0.09	0.09
	RO-IMPT	S-									0.84	0.18	0.03	0.06	0.06
		S+										0.56	0.03	0.06	0.06
		M-											0.03	0.06	0.06
		M+												0.22	0.06
		L-													0.22
		L+													
Brain stem (D2%)		IMRT		0.03	0.03	0.03	0.03	0.03	0.03	0.03	0.03	0.03	0.44	1.00	0.84
	NRO-IMPT	S-			0.83	0.56	0.84	0.69	0.69	0.03	0.03	0.03	0.06	0.09	0.09
		S+				0.06	0.84	0.56	0.69	0.03	0.03	0.03	0.03	0.06	0.06
		M-					0.69	0.56	0.56	0.03	0.03	0.03	0.03	0.03	0.06
		M+						0.22	0.31	0.03	0.03	0.03	0.03	0.06	0.03
		L-							0.16	0.03	0.03	0.03	0.03	0.06	0.06
		L+								0.03	0.03	0.03	0.03	0.06	0.06
	RO-IMPT	S-									0.44	0.40	0.06	0.06	0.16
		S+										0.59	0.03	0.03	0.03
		M-											0.06	0.06	0.09
		M+												0.22	0.44
		L-													0.75
		L+													

				NRO-IMPT						RO-IMPT					
			IMRT	S-	S+	M-	M+	L-	L+	S-	S+	M-	M+	L-	L+
Ipsilateral parotid (Dmean)		IMRT		0.03	0.03	0.03	0.03	0.03	0.03	0.03	0.03	0.03	0.03	0.03	0.03
	NRO-IMPT	S-			0.16	0.56	0.22	0.06	0.06	0.03	0.03	0.03	0.03	0.03	0.03
		S+				0.22	0.56	0.09	0.09	0.03	0.03	0.03	0.03	0.03	0.03
		M-					0.22	0.09	0.06	0.03	0.03	0.03	0.03	0.03	0.03
		M+						0.06	0.03	0.03	0.03	0.03	0.03	0.03	0.03
		L-							0.31	0.03	0.03	0.03	0.03	0.03	0.03
		L+								0.06	0.03	0.03	0.03	0.03	0.03
	RO-IMPT	S-									0.44	0.06	0.03	0.03	0.03
		S+										1.00	0.09	0.06	0.06
		M-											0.03	0.03	0.03
		M+												0.03	0.06
		L-													0.31
		L+													
Contra lateral parotid (Dmean)		IMRT		0.03	0.03	0.03	0.03	0.03	0.03	0.03	0.06	0.03	0.06	0.06	0.06
	NRO-IMPT	S-			0.06	0.56	0.03	0.03	0.03	0.03	0.03	0.03	0.03	0.03	0.03
		S+				0.16	0.31	0.03	0.03	0.03	0.03	0.03	0.03	0.03	0.03
		M-					0.16	0.09	0.03	0.03	0.03	0.03	0.03	0.03	0.03
		M+						0.03	0.03	0.03	0.03	0.03	0.03	0.03	0.03
		L-							0.06	0.06	0.03	0.03	0.03	0.03	0.03
		L+								0.06	0.03	0.06	0.03	0.03	0.03
	RO-IMPT	S-									0.16	0.56	0.03	0.03	0.03
		S+										0.31	0.06	0.03	0.03
		M-											0.03	0.03	0.03
		M+												0.03	0.03
		L-													0.03
		L+													

			IMRT	NRO-IMPT S-	NRO-IMPT S+	NRO-IMPT M-	NRO-IMPT M+	NRO-IMPT L-	NRO-IMPT L+	RO-IMPT S-	RO-IMPT S+	RO-IMPT M-	RO-IMPT M+	RO-IMPT L-	RO-IMPT L+
Oral cavity (Dmean)		IMRT		0.03	0.03	0.03	0.03	0.03	0.03	0.03	0.03	0.03	0.03	0.03	0.03
	NRO-IMPT	S-			0.44	0.56	0.44	0.44	0.44	0.16	0.06	0.16	0.16	0.22	0.16
		S+				1.00	0.69	0.69	0.69	0.06	0.06	0.06	0.09	0.09	0.09
		M-					0.56	0.56	0.56	0.06	0.06	0.06	0.09	0.16	0.16
		M+						1.00	1.00	0.06	0.03	0.03	0.03	0.03	0.03
		L-							0.16	0.22	0.03	0.06	0.03	0.03	0.03
		L+								0.31	0.03	0.06	0.03	0.03	0.03
	RO-IMPT	S-									0.22	0.84	0.44	0.69	0.69
		S+										0.22	0.69	0.69	0.69
		M-											0.56	0.69	0.69
		M+												0.69	0.84
		L-													0.44
		L+													
Larynx (Dmean)		IMRT		0.06	0.03	0.03	0.03	0.03	0.03	0.31	0.84	0.56	0.44	0.56	0.31
	NRO-IMPT	S-			0.84	0.69	0.69	0.22	0.31	0.09	0.06	0.06	0.06	0.06	0.06
		S+				0.84	0.31	0.22	0.06	0.06	0.06	0.06	0.03	0.03	0.06
		M-					0.56	0.44	0.22	0.09	0.06	0.06	0.06	0.06	0.06
		M+						0.22	0.31	0.06	0.06	0.06	0.03	0.03	0.06
		L-							0.44	0.06	0.06	0.06	0.03	0.03	0.06
		L+								0.06	0.06	0.06	0.03	0.03	0.03
	RO-IMPT	S-									0.16	0.31	0.56	0.84	0.31
		S+										0.56	0.31	0.31	0.31
		M-											0.69	0.56	0.56
		M+												0.44	0.31
		L-													0.69
		L+													
