# Supplementary material for: Quantification of beam size impact on intensity-modulated proton therapy with robust optimization in head and neck cancer—comparison with intensity-modulated radiation therapy
Source: J Radiat Res. 2024 Dec 27;66(1):65–73. doi: 10.1093/jrr/rrae097 (PMC11753836; doi:10.1093/jrr/rrae097)
Supplement: Supplement_3_rrae097 [file supplement_3_rrae097.pdf]

Supplement 3
Median values of worst-case target coverage for IMRT, NRO-IMPT, and RO-IMPT. Brackets show dose deviation between nominal and worst-case target coverage.

	Isocenter-shifts / range error	Worst-case dose / (Dose deviation between nominal and worst case)												
		IMRT	NRO-IMPT (Median value %)						RO-IMPT (Median value %)					
			S-	S+	M-	M+	L-	L+	S-	S+	M-	M+	L-	L+
CTV D99%	±1mm / 3%	-	91.5 (-7.2)	92.0 (-7.0)	92.3 (-7.2)	93.1 (-6.4)	93.9 (-7.3)	92.9 (-7.8)	99.6 (-0.9)	99.5 (-1.0)	99.4 (-1.1)	98.8 (-1.5)	98.3 (-2.0)	97.2 (-2.4)
	±2mm / 3%	-	90.0 (-8.5)	90.8 (-8.2)	91.1 (-8.3)	91.7 (-7.7)	92.2 (-9.4)	92.1 (-8.9)	99.3 (-1.2)	99.1 (-1.5)	99.0 (-1.5)	98.3 (-2.0)	97.9 (-2.4)	97.6 (-2.8)
	±3mm / 3%	-	87.1 (-11.0)	88.5 (-10.5)	89.1 (-10.2)	89.8 (-9.7)	89.7 (-11.8)	90.5 (-10.5)	98.8 (-1.8)	98.0 (-2.4)	98.2 (-2.2)	97.2 (-3.3)	96.7 (-3.6)	96.4 (-4.1)
	±4mm / 3%	-	83.8 (-14.3)	85.6 (-13.6)	86.3 (-13.2)	87.8 (-12.0)	87.0 (-14.2)	88.7 (-12.3)	97.5 (-2.9)	97.3 (-3.3)	97.5 (-3.1)	96.0 (-4.4)	96.0 (-4.6)	95.4 (-5.3)
	±5mm / 3%	96.6 (-2.5)	79.0 (-19.0)	82.3 (-17.3)	82.7 (-16.8)	85.3 (-14.8)	84.2 (-16.7)	86.8 (-14.2)	95.6 (-4.8)	95.3 (-5.3)	96.0 (-4.6)	94.3 (-6.2)	94.6 (-6.3)	93.9 (-6.6)
CTV D95%	±1mm / 3%	-	95.2 (-5.1)	95.8 (-5.0)	95.9 (-5.0)	96.5 (-4.6)	97.5 (-4.6)	97.7 (-4.3)	100.7 (-0.6)	100.7 (-0.4)	100.8 (-0.5)	100.8 (-0.5)	101.2 (-0.7)	101.2 (-1.1)
	±2mm / 3%	-	94.3 (-5.8)	95.0 (-5.9)	94.9 (-5.9)	95.8 (-5.5)	96.5 (-5.8)	96.7 (-5.5)	100.5 (-0.8)	100.5 (-0.7)	100.6 (-0.7)	100.6 (-0.8)	101.0 (-1.0)	101.0 (-1.5)
	±3mm / 3%	-	93.2 (-7.0)	93.6 (-6.9)	93.5 (-7.1)	94.6 (-6.8)	95.0 (-7.3)	95.2 (-7.0)	100.2 (-1.0)	100.2 (-1.0)	100.4 (-1.0)	100.3 (-1.0)	100.6 (-1.6)	100.5 (-2.0)
	±4mm / 3%	-	91.4 (-8.8)	92.1 (-8.5)	91.9 (-8.8)	93.6 (-8.1)	93.4 (-8.9)	93.8 (-8.7)	99.9 (-1.4)	100.0 (-1.3)	100.0 (-1.3)	99.7 (-1.7)	100.0 (-2.3)	99.9 (-2.7)
	±5mm / 3%	98.0 (-1.9)	89.7 (-10.5)	90.2 (-10.2)	90.2 (-10.6)	91.5 (-10.2)	91.3 (-11.0)	91.9 (-10.7)	99.4 (-1.9)	99.3 (-1.9)	99.4 (-1.8)	98.9 (-2.5)	98.8 (-3.2)	98.9 (-3.5)
